# Experimental Study of the Jetting Behavior of High-Viscosity Nanosilver Inks in Inkjet-Based 3D Printing

**DOI:** 10.3390/nano12173076

**Published:** 2022-09-05

**Authors:** Xingzhi Xiao, Gang Li, Tingting Liu, Mingfei Gu

**Affiliations:** School of Mechanical Engineering, Nanjing University of Science and Technology, Nanjing 210094, China

**Keywords:** high-viscosity inks, inkjet-based 3D printing, jetting behavior, printable region

## Abstract

Inkjet printing of high-viscosity (up to 10^5^ mPa·s) nanosilver inks is an interesting emerging technology to achieve the 3D fully printed fabrication of electronic products. The highly viscous force of the ink makes it impossible to achieve droplet ejection with the traditional piezoelectric-driven drop-on-demand inkjet method. In this study, a pneumatic needle jetting valve is adopted to provide sufficient driving force. A large number of high-viscosity inkjet printing tests are carried out, and the jetting behavior is recorded with a high-speed camera. Different jetting states are determined according to the recorded images, and the causes of their formation are revealed. Additionally, the effects of the operating pressure, preload angle, and fluid pressure on jetting states are elucidated. Furthermore, the jetting phase diagram is obtained with the characterization of the Reynolds number and the printable region is clarified. This provides a better understanding of high-viscosity inkjet printing and will promote the application of high-viscosity inkjet printing in 3D fully printed electronic products.

## 1. Introduction

Inkjet printing has been developed as a typical 3D printing process method that is usually termed inkjet-based 3D printing. It has attracted wide attention in the application of printed electronics to create functional transistors [1], strains [2], flexible devices [3], RFID tags [4], and antennas [5] due to its potential advantages, such as having a simple process compared with photolithography or etching process, reduction of waste materials, and cost-effectiveness with direct patterning [6]. However, current inkjet-printed electronics are restricted to planar or quasi-3D patterns because the utilized low-viscosity (<1000 mPa·s) conductive ink limits its 3D-forming capability. Vaithilingam et al. [7] explored the fabrication of macroscopic parts via the inkjet printing of low-viscosity silver nanoparticle ink. A 1-mm-high sample was fabricated by printing 1000 layers, which led to a low printing efficiency. In addition, low-viscosity ink is sensitive to the surface quality of a substrate, which may affect the droplet spreading and deposition.

High-viscosity (≥10^5^ mPa·s) conductive ink has been introduced into inkjet-based 3D printing for the 3D fully printed fabrication of electronic products because it is easily compatible with other 3D printing processes to achieve the collaborative printing of both a substrate and a conductive circuit. For example, high-viscosity inkjet printing can be incorporated with fused deposition modeling. The former is utilized to fabricate conductive patterns, and the latter is applied to substructure printing. Furthermore, similar ideas have been reviewed and considered promising technology for customized end-use devices with multifunctionality [8,9]. To realize these ideas, the high-viscosity inkjet printing process should be deeply understood.

High-viscosity inkjet printing requires a larger driving force to form the droplets because of its higher viscous force and surface tension. Pneumatic needles are adopted to provide a sufficient driving force, in contrast to thermal, electrostatic, and piezoelectric driven methods [10,11,12]. The pneumatic needle-driven, drop-on-demand (DOD) printing of high-viscosity inks can be achieved. Additionally, this can overcome many drawbacks that appear in low-viscosity inkjets, such as sensitivity to substrate surface conditions [13], splashing [14], and coffee rings during sintering [15], and it has higher printing efficiency because the layer thickness can be up to 200–1000 μm. However, there are challenges to the printability of high-viscosity ink jetting. For instance, it usually produces a long tail after jetting due to the highly viscous force and the irregular shape of the jetted droplet. Current research on the printability of DOD inkjet printing mainly focuses on low-viscosity inks [16,17,18]. Three important dimensionless numbers, the Reynolds number (*Re*), Weber number (*We*), and Ohnesorge number (*Oh*) are utilized to characterize the physical properties and drop behavior of a droplet during inkjet printing. The specific expressions are as follows [19,20]:

(1) $$Re\;=\;\frac{v\rho d}{\eta }$$

(2) $$We\;=\;\frac{v^{2}\rho a}{\gamma }$$

(3) $$Oh\;=\;\frac{\sqrt{We}}{Re}\;=\;\frac{\eta }{\sqrt{\gamma \rho a}},$$

where *ρ*, *η*, and *γ* are the density, dynamic viscosity, and surface tension of the ink, respectively, v is the velocity, and a is the characteristic length, which is taken as the diameter of the printer orifice [20].

From [21] proposed the number *Z* (*Z = 1/Oh*) in his study and pointed out that *Z* > 2 for the obtainment of a stable drop as the lower value of *Z* results in a large viscous force that hindered the droplet formation [22]. Reis and Derby [23] explored ink jetting behavior by using computational fluid dynamics (CFD) modeling and experimental study, and they found that 1 < *Z* < 10 was the printable region. When *Z* < 1, the viscous dissipation prevented drop formation, whereas when *Z* > 10, many satellite droplets were generated along with the primary droplet. Jang et al. [24] experimentally defined the printable range as 4 ≤ *Z* ≤ 14. With a low *Z* value (*Z* < 4), the droplet formed with a long-lived filament, whereas at a high *Z* value, a single droplet could not be formed due to satellite droplets. To overcome the ink surface tension at the printer orifice, the minimum velocity for the droplet ejection was defined by Duineveld et al., and it is represented as the following minimum Weber number [25],

(4) $$We_{min}\;=\;v_{min}{\left(\frac{4\gamma }{\rho a}\right)}^{1/2}>4.$$

When considering the splashing that occurred during the droplet impact with the substrate, the maximum velocity can be limited by the formula proposed by Stow and Hadfield [26],

(5) $$We^{1/2}Re^{1/4}\;=\;K\left(r\right),$$

where *K(r)* is the function of the substrate roughness.

Liu and Derby [22] determined the jet Weber number *We_j_* by using the velocity of the ink prior to drop formation and defined the printable region as 2 < *We_j_* < 25 and 2 < *Z* < 20. The lower limit of *We_j_* represents the limiting capillarity forces that must be overcome for drop formation. The upper limit of *We_j_* represents the intrinsic instability of the extended tail that forms as a drop is ejected. Zhao et al. [27] also proposed a process dynamic–related dimensionless number *Wj* to construct a phase diagram for droplet generation.

The above studies have shown that the drop formation principle and the printable region are relatively well understood for low-viscosity inkjet printing. However, this principle and printable region are not suitable for high-viscosity inks. Low-viscosity inks are considered to be Newtonian fluids and are incompressible, whereas high-viscosity inks are typical non-Newtonian fluids and present shear thinning characteristics. Additionally, for a typical high-viscosity nanosilver ink with a viscosity of 10^5^ mPa·s, the velocity of the formed droplet in the printer orifice is in the range of 10 m/s to 170 m/s via pneumatic needle-driven jetting, the calculated We number is larger than 1000, and the *Z* number is about 0.3 with consideration of the shear thinning. These values are beyond the printable region obtained in the above studies, but in reality, a single droplet can be formed under this condition [28]. Thus, there is an urgent need for further investigation of the physical properties and droplet behavior of ink for high-viscosity inkjet printing.

In this study, a large number of tests are conducted to study the droplet ejection of high-viscosity nanosilver inks during pneumatic needle-driven jetting. A high-speed camera is utilized to capture the droplet morphology and movement conditions. The jetting behaviors are analyzed and the printable phase diagram is given. This study will help broaden the range of material adaptations for inkjet printing and provide a better understanding of the control of jetting states.

## 2. Materials and Methods

### 2.1. Experimental Material

**Figure 1:**
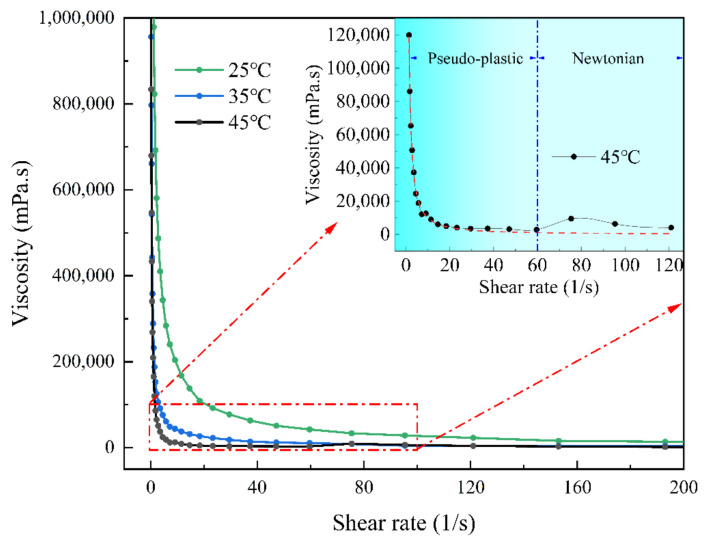
Effect of shear rate and temperature on viscosity [28].

To investigate the droplet behavior of high-viscosity inks in inkjet printing, a nanosilver ink (NT-ST60S, Nano Top Co., Ltd., Beijing, China) with a viscosity of approximately 10^5^ mPa·s and a density of 2.3 g/cm^3^ is selected for the jetting experiments. The nanosilver paste is a non-Newtonian fluid and exhibits shear-thinning and temperature-dependent characteristics. A dynamic shear rheometer (MCR-92, Anton Paar GmbH, Graz, Austria) is utilized to obtain the viscosity for varied shear rates and temperatures. As shown in Figure 1, the viscosity decreases with the rise of the shear rate, and the higher temperature results in lower viscosity. In this study, the nozzle temperature is fixed at 45 °C and the viscosity after shear-thinning is considered to remain constant, as shown in the enlarged figure in Figure 1.

### 2.2. Experimental Setup

**Figure 2:**
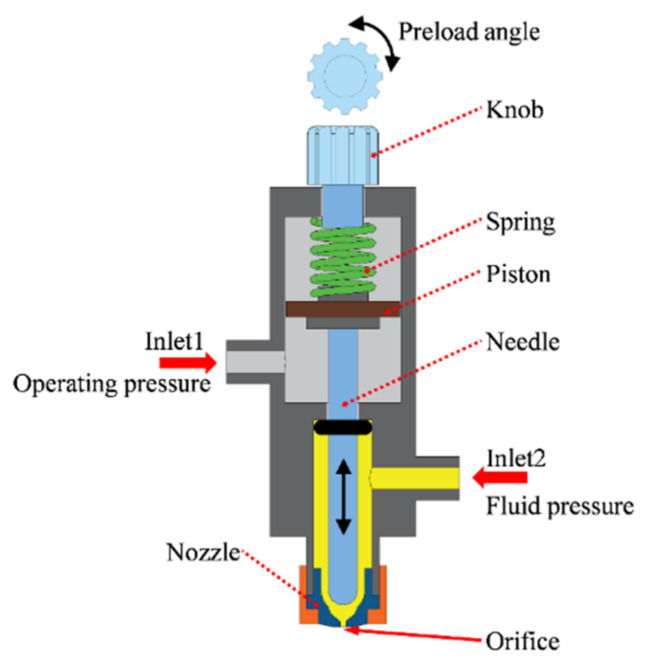
Schematic illustration of the pneumatic needle jetting valve.

The pneumatic needle jetting valve is used for high-viscosity nanosilver inks because it can provide a high-impact force to overcome the large viscosity force of the inks. As shown in Figure 2, the valve consists of a nozzle, a needle, a reservoir, a piston, a spring, and a knob. When the compressed air enters via inlet 2, the operating pressure acts on the piston and pushes the piston upward, which results in the compression of the spring and the lift of the needle. In this condition, the fluid pressure acts on the high-viscosity nanosilver ink and the ink fills the gap between the needle and the nozzle. When the operating pressure is removed, the needle moves downward, driven by the compressed spring, and hits the nozzle, creating a huge impact force that prompts the high-viscosity ink to leave the orifice and form the droplet. The top knob is used to adjust the preload of the spring, which affects the initial gap between the needle and the inner wall of the orifice as well as the ultimate impact force. A preload angle of 0° means that the end of the needle has just come into contact with the inner wall of the orifice. When the preload angle is less than 0°, it represents the fact that there is a gap between the needle and the inner wall, and if the preload angle is greater than 0°, it means that there is already pressure between the needle and the inner wall.

### 2.3. Experimental Design

The preload angle, operating pressure, and fluid pressure are selected as input variables. Each variable has nine levels. The specific values are listed in Table 1. The specific parameter combinations are determined via full-factorial experimental design, with a total of 729 sets of experiments. For each parameter combination, five jetting tests are performed to obtain stable conditions.

### 2.4. Evaluation Methods

The formation and the flight of the high-viscosity droplet are captured by a high-speed camera (X213, FuHuang Agile Device Co., Ltd., Hefei, China). The frame rate used is 100,000 fps with a shutter speed of 2/1,000,000 s, and the resolution is 800 × 128 pixels. The captured pictures are used to analyze the droplet behavior and to calculate the flight velocity of the droplet, particularly for the outlet velocity of the droplet.

## 3. Results and Discussion

### 3.1. Jetting State Analysis for High-Viscosity Inkjet Printing

**Figure 3:**
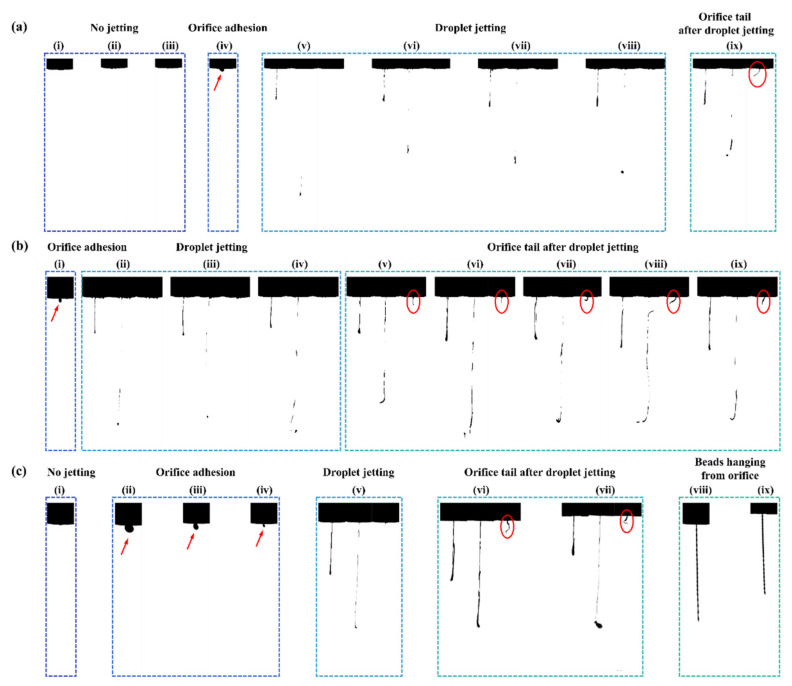
Jetting states with different input variables. (**a**) Operating pressure varying from 2 bar to 5.2 bar with increments of 0.4 bar (corresponding to (**i**) to (**ix**)) and the preload angle and fluid pressure fixed at −90° and 3.6 bar, respectively. (**b**) Fluid pressure varying from 2 bar to 5.2 bar with increments of 0.4 bar (corresponding to (**i**) to (**ix**)) and the preload angle and operating pressure fixed at −90° and 4.8 bar, respectively. (**c**) Preload angle varying from −180° to 180° with increments of 45° (corresponding to (**i**) to (**ix**)) and the operating pressure and fluid pressure fixed at 4.0 bar and 5.2 bar, respectively.

The jetting process, from high-viscosity ink reaching the nozzle orifice to droplet flight, is recorded with a high-speed camera. However, it is unrealistic to show the images of all 729 sets of jetting processes at the same time. For each input variable, a group of images containing nine different levels is selected to analyze the jetting state for high-viscosity inkjet printing, as shown in Figure 3.

Figure 3a shows the jetting state at different operating pressures, with the preload angle and the fluid pressure remaining constant at −90° and 3.6 bar, respectively. It is found that there are four different jetting states, namely no jetting, orifice adhesion, droplet jetting, and orifice tail after droplet jetting. Figure 3b shows the jetting state at different fluid pressures, with the preload angle and the operating pressure remaining constant at −90° and 4.8 bar, respectively. Orifice adhesion, droplet jetting, and orifice tail after droplet jetting are also found. Figure 3c shows the jetting state at different preload angles, with the operating pressure and the fluid pressure remaining constant at 4.0 bar and 5.2 bar. No jetting, orifice adhesion, droplet jetting, orifice tail after droplet jetting, and beads hanging from the orifice are found in this circumstance. To summarize, five jetting states, no jetting, orifice adhesion, droplet jetting, orifice tail after droplet jetting, and beads hanging from orifice, can be found in high-viscosity inkjet printing. This shows different jetting states compared with a low-viscosity inkjet. Liu et al. [22] found that a single droplet, satellite coalescence, one stable satellite, and two or more satellites existed for a low-viscosity inkjet. For a high-viscosity inkjet, the jetting process becomes more complicated with the impact of the needle. The ejected high-viscosity ink is asymmetrical in shape and it is not similar to the teardrop shape that appears in a low-viscosity inkjet. Additionally, it is almost impossible to find a single droplet jetting state during flight, and the ejected ink basically contains multiple satellite droplets. For the selected nanosilver ink, due to the high solids content (accounting for more than 70 wt%) and viscosity, the silver particles are unevenly dispersed in the material, and the force state of the ejected ink is asymmetrical, resulting in an irregular shape of the ejected high-viscosity ink. In addition, the impact of the pneumatic needle gives the ejected drop head a high velocity and the high viscous force makes it difficult for the main droplet to break, resulting in a long tail behind the main drop. The higher velocity of the drop head compared with the tail causes the breakdown of the tail into small fractions and the formation of satellite droplets [11]. Therefore, the simultaneous high flight velocity and the viscous force of the ejected ink are the main reason why high-viscosity inkjet printing tends to produce satellite droplets.

Compared with low-viscosity inkjet printing, there is a needle in the reservoir and the needle strikes the inner wall of the nozzle orifice, providing the initial kinetic energy to the ink under the needle. The ink that obtains the initial kinetic energy has a tendency to move downwards. If the inertial force of the ink is less than the viscous force, no liquid is ejected out of the nozzle orifice and it is considered that no jetting has occurred, as shown in Figure 3a(i–iii),c(i). If the inertial force is greater than the viscous force, the ink can be ejected out of the orifice, but the final jetting state is determined by the kinetic energy of the ejected ink and the initial position of the needle.

When the kinetic energy of the ejected ink cannot overcome the work done by the viscous force, the ejected ink does not break to form droplets. Under this condition, if there is a gap between the needle and the inner wall of the orifice in their initial positions (if the preload angle is less than 0°), the ink is more similar to flow out of the orifice due to the absence of the needle cutting effect. The outflow ink will adhere to the outer wall of the orifice and the volume of the adhesive ink will gradually increase with subsequent jetting. This jetting state is called orifice adhesion, as shown in Figure 3a(iv),b(i),c(ii–iv) with red arrows. If the needle comes into contact with the inner wall of the orifice in the initial positions (if the preload is equal to or greater than 0°), the ejected ink will form a liquid bead and hang from the orifice due to the cutting effect of the needle. With the subsequent ejection, a string of liquid beads will be formed under the orifice. This jetting state is called beads hanging from the orifice, as shown in Figure 3c(viii–ix).

When the kinetic energy of the ejected ink is sufficient to overcome the work done by the viscous force, the ejected ink can break and form droplets regardless of the initial position of the needle. Then the jetting state is called droplet jetting. In this case, the velocity at which the needle hits the inner wall of the orifice downward is generally larger, and the excessive velocity of the needle will rebound after hitting the inner wall of the orifice, forming a spontaneous second impact. If the second spontaneous impact can still cause the ink to eject but not break to form new droplets, a tail will be formed at the orifice, which is called an orifice tail after droplet jetting. The residual tails are shown in the circles in Figure 3a(ix),b(x–ix),c(vi–vii).

### 3.2. Effect of Input Variables on Droplet Behavior

**Figure 4:**
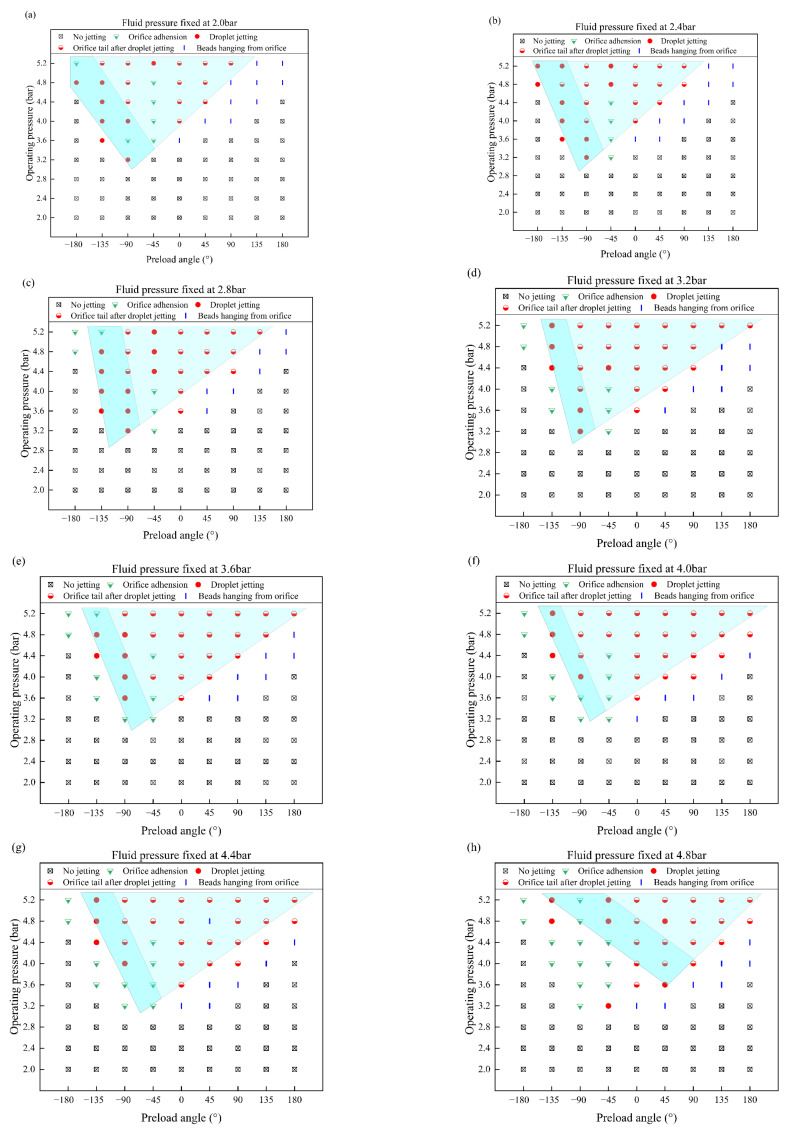

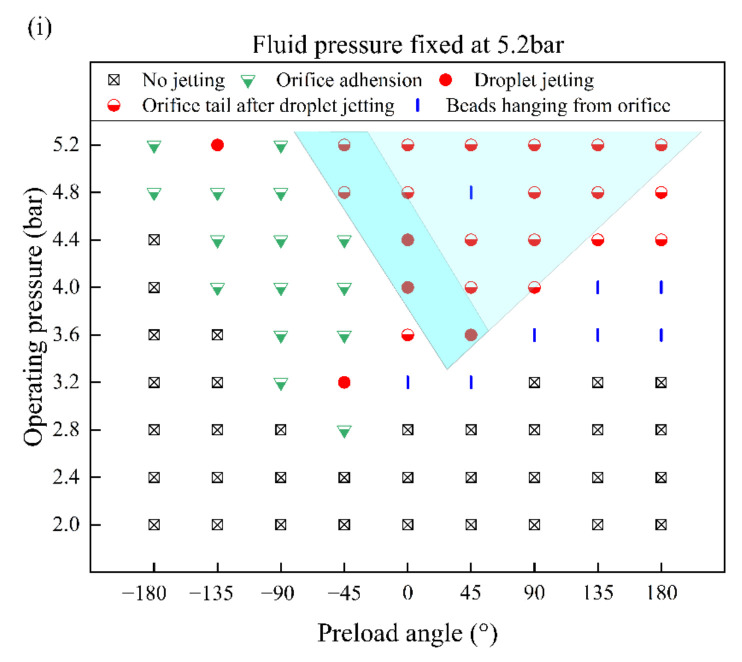
Jetting states under different preloading angles and operating pressures with the fluid pressure fixed at values of (**a**) 2.0 bar, (**b**) 2.4 bar, (**c**) 2.8 bar, (**d**) 3.2 bar, (**e**) 3.6 bar, (**f**) 4.0 bar, (**g**) 4.4 bar, (**h**) 4.8 bar, and (**i**) 5.2 bar.

The operating pressure, preload angle, and liquid pressure are important input variables that have a significant effect on the jetting states in high-viscosity inkjet printing. The effects of these variables on the jetting states are revealed in the analysis of the images captured during jetting, as shown in Figure 4.

The abscissa of the figure is the preload angle and the ordinate is the operating pressure. Figure 4a–i represents the jetting state with different fluid pressures. In Figure 4, the squares with cross symbols inside indicate that no jetting has occurred. The semi-filled inverted triangles represent orifice adhesion. The filled circles indicate the ejection of droplets. The semi-filled circles represent the formation of a tail outside the nozzle orifice after droplet jetting. The short vertical bars indicate the presence of beads hanging from the orifice without droplet jetting. The blue-filled polygon area identifies the printable region with different input parameters. The dark blue area represents droplet jetting and the light blue area represents the orifice tail after droplet jetting.

In general, the shape of the printable regions with different fluid pressures is similar to an inverted triangle. According to the structure of the pneumatic needle jetting valve and the definition of the preload mentioned previously, the downward velocity of the needle gradually increases as the preload angle increases from −180° to +180°. However, the kinetic energy of the ink that is about to be jetted through the orifice does not gradually increase because the excessive needle velocity causes some of the ink under the needle to flow upward along the inner wall of the orifice, which consumes some of the energy, resulting in a decrease in the initial kinetic energy obtained by the ink that is about to be jetted through the orifice. Additionally, the greater the needle velocity is, the more significant is the tendency of the ink to flow upward along the inner wall of the orifice. This phenomenon was found by Li via CFD simulation [28]. Therefore, as the preload angle increases, it gradually changes from the no-jetting state to the printable state, but when the preload angle is too large, beads hang from orifice and no jetting can occur. Finally, this results in an inverted triangular printable region.

Comparing Figure 4a–i, it can be seen that the inverted triangular printable region gradually moves from left to right with the increase of the fluid pressure. When the preload angle is small (<0°), the upward flow of the ink under the needle is not obvious. The increase in the fluid pressure leads to an increase in the volume of the ink to be ejected, which results in a decrease in the velocity of the ink after the impact of the needle. Hence, the kinetic energy of the ejected ink is not enough to overcome the work done by the viscous force and the ink cannot break to form droplets, and the orifice adhesion state occurs. When the preload angle is large (>0°), the needle velocity is large enough to cause the ink to move upward along the inner wall of the orifice. The increase in the fluid pressure can effectively suppress the upward flow of the ink and reduce the energy loss so that the kinetic energy of the ejected ink is sufficient to overcome the work done by the viscous force and droplet jetting appears. Therefore, the inverted triangular printable region moves from left to right with the increase of the fluid pressure.

In terms of operating pressure, its increase makes the distance of the needle move upward, and the needle velocity and the ink volume under the needle increase at the same time, resulting in more energy being transferred to the ink. Thus, it can realize the transformation of the jetting state from no jetting to jetting without droplets (orifice adhesion or beads hanging from orifice), and then to droplet jetting. Additionally, there is a critical operating pressure to obtain the jet droplets. As shown in Figure 4, the critical operating pressure is 3.2 bar. When the operating pressure is lower than 3.2 bar, no jet droplets can be formed regardless of the preload angle or fluid pressure. The critical operating pressure represents the minimum inertial force required for droplet formation.

Through further analysis, it is found that the orifice adhesion phenomenon mainly exists in the range of a preload angle of less than 0°, whereas the beads hanging from the orifice mainly exist in the range of a preload angle of greater than 0°. This is consistent with the analysis of the causes of orifice adhesion and beads hanging from orifices, which has been discussed previously.

Furthermore, as can be seen from Figure 4a–i, when the preload angle is −45°, the ejected ink is easy to adhere to the outer wall of the orifice and form orifice adhesion. At a certain operating pressure and when the preload angle is less than zero, the potential energy of the needle in the initial state gradually increases with the increase of the preload angle, and the overall velocity at which the needle moves downward also gradually increases. During the downward movement of the needle, the ink under the needle obtains kinetic energy for downward and peripheral movement. When the needle moves downwards faster, the ink does not have enough time to move toward the periphery. As a result, most volumes of the ink under the needle acquires potential energy for downward motion. This volume of the ink consumes more energy as it passes through the orifice, resulting in less kinetic energy of the ejected ink. At this time, the kinetic energy of the ejected ink is not enough to overcome the work done by the viscous force; thus, the independent droplets cannot be formed, and the ejected ink easily adheres to the outer wall of the orifice to form orifice adhesion. Therefore, the droplet jetting can be formed at a preload angle of −90° while the orifice adhesion is easy to form at a preload angle of −45°.

**Figure 5:**
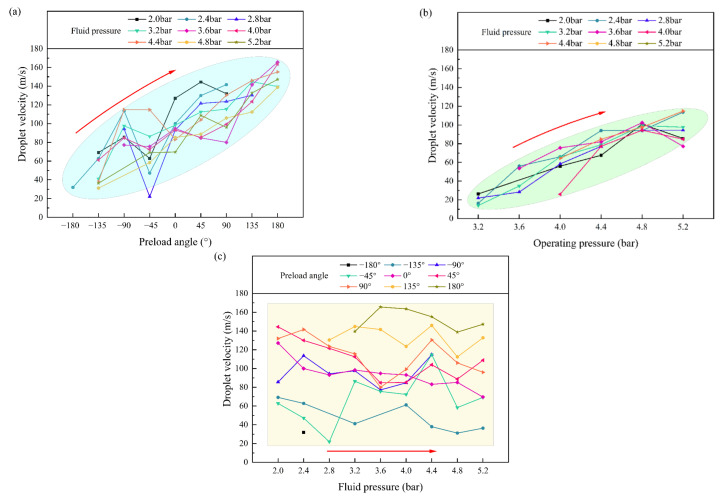
Effect of (**a**) preload angle, (**b**) operating pressure, and (**c**) fluid pressure on drop velocity.

Figure 5 shows the effects of the operating pressure, preload angle, and fluid pressure on droplet outlet velocity. Because the velocity is calculated according to the images captured by the high-speed camera, only the velocity of droplets in the printable region (droplet jetting and orifice tail after droplet jetting states) can be obtained. The droplet velocity gradually increases with the increase of the preload angle and the operating pressure, but the effect of the preload angle is significantly greater, as shown in Figure 5a,b. The droplet velocity fluctuates with the increase of the fluid pressure, which indicates that the fluid pressure has less influence on the droplet velocity, as shown in Figure 5c. This is consistent with the contour and changing trend of the printable region, thus supporting the reliability of the obtained printable region.

**Figure 6:**
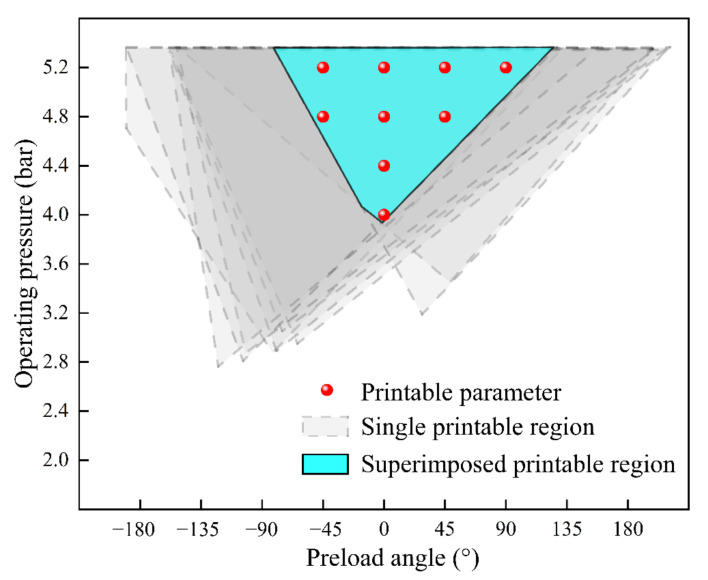
Printable region after superimposition.

The jetting states of Figure 4a–i are superimposed to obtain a more detailed printable region regardless of the influences of the fluid pressure. In Figure 6, the light blue area is the superimposed printable region, and the light gray dotted areas represent the printable region under different fluid pressures. The figure shows that the fine printable area is also an inverted triangle with an operating pressure between 4 bar and 5.2 bar and a preload angle between −45° and 90°.

### 3.3. The Phase Diagram of the Jetting Behavior

**Figure 7:**
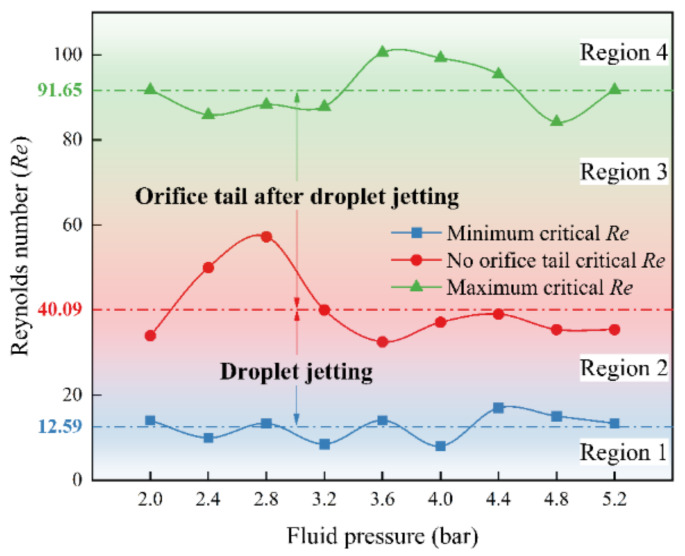
Jetting phase diagram characterized by Reynolds number.

During high-viscosity inkjet printing, the liquid velocity varies from 10 m/s to 170 m/s, and the inertial force and the viscous force related to the liquid velocity have significant effects on the jetting state, whereas the surface force independent of the velocity is relatively low and has a limited effect on the jetting state. When discussing the effects of the inertial force and the viscous force on the jetting state, it is found that the ratio of the inertial force to the viscous force, i.e., the Reynolds number, can be used to characterize the jetting state. Therefore, the jetting phase diagram can be obtained more concisely and clearly with the Reynolds number, as shown in Figure 7. Figure 7 is divided into four regions according to the value of the Reynolds number. Region 1 (*Re* < 12.59) represents no jetting and orifice adhesion. Region 2 (12.59 ≤ *Re* < 40.09) denotes droplet jetting. Region 3 (40.09 ≤ *Re* < 91.65) indicates the orifice tail after droplet jetting. Region 4 (*Re* > 91.65) represents beads hanging from the orifice without a droplet. Additionally, it is found that the effect of the fluid pressure on the Reynolds number is limited because the fluid pressure mainly presses the ink from the syringe into the reservoir and has less impact on the outlet velocity of the drop. However, it can also be seen that when the Reynolds number value is larger, the fluctuation of the Reynolds number increases as the fluid pressure changes. The main reason for this is that the effect of fluid pressure on the upward flow of the ink along the inner wall of the orifice is intensified in the case of a larger Reynolds number, and this results in the fluctuation of the droplet outlet velocity.

## 4. Conclusions

In this study, a large number of experiments are carried out for high-viscosity nanosilver inks during inkjet-based 3D printing. The jetting behavior is captured via a high-speed camera and the droplet outlet velocity is calculated according to the captured images. The jetting states and the printable region are determined. The following conclusions can be drawn.

(1) Compared with low-viscosity inkjet printing, high-viscosity inkjet printing shows different jetting behaviors. Five different jetting states are clarified, which are no jetting, orifice adhesion, droplet jetting, orifice tail after droplet jetting, and beads hanging from an orifice.
(2) The inverted triangular printable region is determined and the printable region moves from left to right in the direction of the increase of fluid pressure. The effects of the preload angle, fluid pressure, and operating effect on the jetting state are elucidated. Additionally, a detailed printable region is obtained with superimposition.
(3) By using the Reynolds number, a concise and clear jetting phase diagram is obtained. The printable region is bounded by 12.59 ≤ *Re* < 91.65.

This study provides a comprehensive and detailed investigation into the jetting behavior of high-viscosity inkjet printing and contributes to a better understanding of high-viscosity inkjet printing.

## Figures and Tables

**Table 1:** Values of the input variables.

Preload Angle/°	Operating Pressure/Bar	Fluid Pressure/Bar
−180, −135, −90, −45, 0, 45, 90, 135, 180	2, 2.4, 2.8, 3.2, 3.6, 4.0, 4.4, 4.8, 5.2	2, 2.4, 2.8, 3.2, 3.6, 4.0, 4.4, 4.8, 5.2

## Data Availability

Not applicable.
